# Detection of an inversion in the *Ty*-*2* region between *S. lycopersicum* and *S. habrochaites* by a combination of de novo genome assembly and BAC cloning

**DOI:** 10.1007/s00122-015-2561-6

**Published:** 2015-07-08

**Authors:** Anne-Marie A. Wolters, Myluska Caro, Shufang Dong, Richard Finkers, Jianchang Gao, Richard G. F. Visser, Xiaoxuan Wang, Yongchen Du, Yuling Bai

**Affiliations:** Wageningen UR Plant Breeding, Wageningen University & Research Centre, P.O. Box 386, 6700 AJ Wageningen, The Netherlands; The Institute of Vegetables and Flowers, Chinese Academy of Agricultural Sciences, Zhongguancunnandajie 12, Beijing, 100081 People’s Republic of China

## Abstract

**Key message:**

**A chromosomal inversion associated with the tomato*Ty*-*2*gene for TYLCV resistance is the cause of severe suppression of recombination in a tomato*Ty*-*2*introgression line.**

**Abstract:**

Among tomato and its wild relatives inversions are often observed, which result in suppression of recombination. Such inversions hamper the transfer of important traits from a related species to the crop by introgression breeding. Suppression of recombination was reported for the TYLCV resistance gene, *Ty*-*2,* which has been introgressed in cultivated tomato (*Solanum lycopersicum*) from the wild relative *S. habrochaites* accession B6013. *Ty*-*2* was mapped to a 300-kb region on the long arm of chromosome 11. The suppression of recombination in the *Ty*-*2* region could be caused by chromosomal rearrangements in *S. habrochaites* compared with *S. lycopersicum.* With the aim of visualizing the genome structure of the *Ty*-*2* region, we compared the draft de novo assembly of *S. habrochaites* accession LYC4 with the sequence of cultivated tomato (‘Heinz’). Furthermore, using populations derived from intraspecific crosses of *S. habrochaites* accessions, the order of markers in the *Ty*-*2* region was studied. Results showed the presence of an inversion of approximately 200 kb in the *Ty*-*2* region when comparing *S. lycopersicum* and *S. habrochaites*. By sequencing a BAC clone from the *Ty*-*2* introgression line, one inversion breakpoint was identified. Finally, the obtained results are discussed with respect to introgression breeding and the importance of a priori de novo sequencing of the species involved.

**Electronic supplementary material:**

The online version of this article (doi:10.1007/s00122-015-2561-6) contains supplementary material, which is available to authorized users.

## Introduction

In genetics, introgression (also known as introgressive hybridization) is the transfer of a gene from one species into the gene pool of another species. Such a transfer starts with an interspecific hybridisation and is followed by backcrossings with one of the parental species. In breeding, introgression is an important strategy to broaden the genetic base of highly inbred crops such as tomato by transferring economically important traits from a related species to the crop. One of the major problems in introgression breeding is caused by chromosomal rearrangements, such as inversions and translocations, between the donor species and the crop (Szinay et al. 2010). Genetic maps created from different intraspecific or interspecific crosses using the same markers can indicate co-linearity or a change in order of markers in distinct chromosomal regions. In the Solanaceae family, genetic maps have been used to detect chromosomal rearrangements (e.g. Wu and Tanksley 2010; Doğanlar et al. 2014). Also, the study of pachytene synaptonemal complexes in interspecific F_1_ hybrids can indicate the presence of chromosomal rearrangements (Anderson et al. 2010). Meanwhile, cross-species BAC fluorescence in situ hybridisation (FISH) analysis has been shown to be a powerful instrument to identify chromosomal rearrangements in the Solanaceae family (van der Knaap et al. 2004; Iovene et al. 2008; Tang et al. 2008) and more specifically, among related species of *Solanum* (Lou et al. 2010; Verlaan et al. 2011; Peters et al. 2012; Szinay et al. 2012; Shearer et al. 2014). In introgression breeding this technology can be used as a diagnostic tool to monitor meiotic disturbances in the pairing of homoeologous chromosomes from crops and their related species. Nowadays, the released full genome sequences of closely related species have facilitated comparative genome analysis. Occurrences of both large-scale and small-scale rearrangements have been reported between tomato and potato genomes (The Potato Genome Sequencing Consortium 2011; The Tomato Genome Consortium 2012). For a co-linearity study of two genomes, a reference genome sequence should not only be available for the cultivated species, but also for the wild donor species. To this end, de novo assembly of genome sequences of three wild relatives of tomato has been undertaken recently (The 100 Tomato Genome Sequencing Consortium 2014).

Among tomato and its wild relatives, inversions are often observed (Szinay et al. 2012) which can cause meiotic pairing disturbances between homoeologues. Crossovers are unlikely to occur in the inverted region, which results in suppression of recombination (Szinay et al. 2010). Thus, the inverted region will be genetically inherited as one locus during the introgression and many unwanted sequences in the inverted region will be transferred together with the gene of interest from the wild donor to the crop species, a phenomenon known as linkage drag. A good example is the *Ty*-*1* gene which originated from *S. chilense* and confers resistance to Tomato yellow leaf curl virus (TYLCV) (Verlaan et al. 2013). Chromosomal rearrangements between *S. chilense* and the cultivated tomato were detected by BAC-FISH (Verlaan et al. 2011). These rearrangements caused severe suppression of recombination in the *Ty*-*1* region and thus hampered the *Ty*-*1* introgression (Verlaan et al. 2011). Suppression of recombination was reported for another TYLCV resistance gene, *Ty*-*2,* which has been introgressed in cultivated tomato (*Solanum lycopersicum*) from the wild relative *S. habrochaites* accession B6013 (Kalloo and Banerjee 1990). The gene has been mapped on the long arm of chromosome 11 (Hanson et al. 2000; Ji et al. 2009), and fine mapped to a 300-kb region (Yang et al. 2014). Attempts to clone the gene have been hampered by the occurrence of severe suppression of recombination in a large part of this region. Such suppression of recombination could be caused by chromosomal rearrangements in *S. habrochaites* compared with *S. lycopersicum,* as shown previously for the *Ty*-*1* gene (Verlaan et al. 2011). However, for the *Ty*-*2* region, FISH on pachytene chromosomes using three BACs spanning the introgression region and one BAC outside the region resulted in overlapping fluorescing signals (Yang et al. 2014). Because of this, the order of the BACs could not be determined, and therefore no conclusion could be drawn on the cause of the suppression of recombination.

In order to visualize the genome structure of the *Ty*-*2* region, in this study we combined de novo genome assembly and BAC cloning. First, the draft de novo assembly of *S. habrochaites* accession LYC4 was compared with the genome sequence of cultivated tomato (‘Heinz’) to determine whether a chromosomal rearrangement has occurred in the *Ty*-*2* region. Secondly, BAC cloning of the *Ty*-*2* introgression line was performed. Furthermore, recombinant screening of F_2_ populations derived from intraspecific crosses of *S. habrochaites* accessions was carried out. Taken together, the results showed the presence of an inversion of approximately 200 kb in the *Ty*-*2* region when comparing *S. lycopersicum* and *S. habrochaites*.

## Materials and methods

### Plant materials and DNA isolation

*Solanum lycopersicum* ‘Moneymaker’ (MM), *S. habrochaites* accessions LYC4, G1.1560 (=CGN15790), G1.1257 (=CGN15370), G1.1606 (=CGN24036) and G1.1290 (=CGN15391) were obtained from the Centre for Genetic Resources (CGN), Wageningen, the Netherlands.

F_2_ family PV95279 was obtained from an interspecific cross between *S. lycopersicum* MM and *S. habrochaites* accession G1.1257. F_2_ families of intraspecific crosses between *S. habrochaites* accessions were PV960350 (G1.1257 × G1.1560), PV960357 (G1.1560 x G1.1606) and PV970303 (G1.1290 × G1.1560). Seeds from the F_2_ populations and parental accessions were sown in plastic cell trays and kept in a germination chamber for germination. The temperature of this chamber was between 25 and 27 °C with 90 % relative humidity. Genomic DNA was extracted from 2- to 3-week-old seedlings using a cetyltrimethyl ammonium bromide (CTAB) protocol (Fulton et al. 1995).

### Sequence alignment

The *S. habrochaites* LYC4 sequence from the *Ty*-*2* region was extracted from the draft de novo assembly (The 100 Tomato Genome Sequencing Consortium 2014). Pairwise comparisons between sequences were made using the WebAct tool (Abbott et al. 2005) using default settings. Resulting alignments were visualized using the Artemis comparison tool ACT (Carver et al. 2005) with the footprint slider set at 101 (filter the regions of similarity based on the length of sequence over which the similarity occurs). The dot plot was obtained by aligning the *Ty-2* regions from *S. lycopersicum* ‘Heinz’ and *S. habrochaites* LYC4 using MAFFT version 7 (http://mafft.cbrc.jp/alignment/server/, Katoh and Standley 2013). Sequence analyses were performed with DNASTAR Lasergene 8 and Vector NTIAdvance 11 (Invitrogen).

### Recombinant screening

CAPS markers used for fine-mapping of the *Ty*-*2* region (Ji et al. 2009; Yang et al. 2014) were tested for polymorphisms between different *S. habrochaites* accessions. PCR products obtained from the different accessions were sequenced, and the sequences were aligned to discover SNPs. When possible, co-dominant CAPS markers were developed to distinguish the different parental alleles. Primer sequences are presented in Table S1. PCRs were performed in 96-wells plates. PCR products were digested with restriction enzymes from Thermo Scientific and New England Biolabs.

### Construction and screening of BAC library

The cultivated tomato line 12 g-60, homozygous for the smallest introgression containing the *Ty*-*2* resistance gene from *Solanum habrochaites* B6013, was selected for the construction of a bacterial artificial chromosome (BAC) library. *Hin*dIII fragments were cloned into vector CopyControl™ pCC1BAC™ (*Hin*dIII Cloning-Ready) (Epicentre), and transformed to *E. coli* strain TransforMax™ EPI300™ (Epicentre), according to a previously described protocol (Rouppe van der Voort et al. 1999). The BAC library consisted of 99,840 clones with an average insert size of 100 kb, corresponding to 10 times coverage of the tomato genome. The library was stored in 260 384-well microtiter plates, and all 384 clones in one plate were mixed to form a BAC pool. The BAC pool DNA was isolated by alkaline lysis method and screened by PCR using 17 primer pairs within and flanking the *Ty*-*2* region (Table S2). Afterwards, individual colonies from the 384-well plates corresponding to the positive BAC pools were identified using the same markers, and DNA was isolated from the positive colonies.

### DNA sequencing and analysis

BAC ends were sequenced to confirm that they originated from the *Ty*-*2* region. Complete sequences of the selected BAC clones (16-100 kb) were obtained by constructing a library of subclones (1–3 kb). Both ends of the subclones were sequenced using the ABI 3730xl platform and then assembled (BGI, Beijing, China). Putative genes in the BAC sequence were predicted with the online Softberry program FGENESH (Solovyev et al. 2006). Results were compared with the ‘Heinz’ 1706 genome annotations derived from the International Tomato Annotation Group (ITAG2.3 version). Primers used to analyse the putative inversion breakpoints were bpTyF1 (5′-AAACTCACACCGCTCCGTTGTC-3′), bpTyR1 (5′-CCTCTTCCGATCTTTGGGTACA-3′) and bpTyR2 (5′-TGTTGGCATGTGACTTATAGGTA-3′).

## Results

### Bioinformatic comparison of the *Ty*-*2* region from *S. lycopersicum* and *S. habrochaites*

**Fig. 1:**
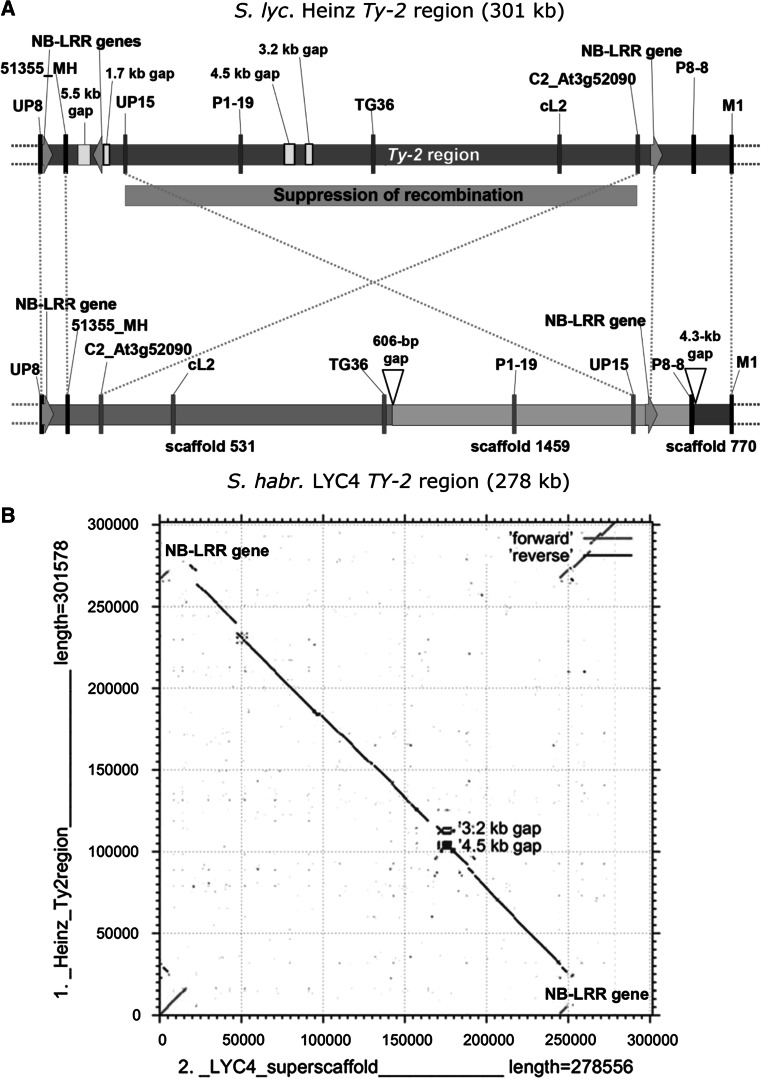
Comparison between the *Ty*-*2* genomic region of *S. lycopersicum* ‘Heinz’ and the superscaffold spanning the *Ty*-*2* region in *S. habrochaites* LYC4 (**a**) Visual representation of the *Ty*-*2* regions in ‘Heinz’ and LYC4. Markers in the ‘suppression of recombination’ block are indicated in *red* (UP15, P1-19, TG36, cL2 and C2_At3g52090), and the other markers are in *black* (UP8, 51355_MH, P8-8 and M1). Gaps in the ‘Heinz’ sequence are shown in *light blue bars* and the sizes of these gaps are estimated by the number of “N” in the tomato genome. NB-LRR genes are indicated as *green arrows*. *Orange dotted lines* connect homologous sequences in LYC4 compared with ‘Heinz’. **b** Dot plot of the alignment of the *Ty*-*2* regions of ‘Heinz’ and LYC4. *Red lines* indicate co-linearity; *blue lines* indicate inversion. The gaps in the ‘Heinz’ sequence disrupt the co-linearity of the two sequences

The region containing the *Ty*-*2* resistance gene was determined to span a 300-kb sequence at the distal end of the long arm of chromosome 11, flanked by markers UP8 and M1 (Fig. 1a, Yang et al. 2014). This corresponds to the region between nucleotides 51,344,943 and 51,646,517 on chromosome 11 of tomato genome version SL2.40, or between nucleotides 54,261,443 and 54,563,017 on chromosome 11 of tomato genome version SL2.50. We prefer to use the coordinates of the SL2.40 version in this paper for easy reference to previous articles.

A BLAST analysis of this region was performed against the draft de novo assembly of the *S. habrochaites* LYC4 genome. Three large scaffolds (531, 1459 and 770) spanned most of the *Ty*-*2* region (Fig. 1a). Interestingly, *S. habrochaites* LYC4 scaffold 531 contained both the flanking marker UP8 and marker C2_At3g52090 at a distance of only 26 kb, whereas in *S. lycopersicum* ‘Heinz’ the distance between these two markers is 262 kb. Additionally, *S. habrochaites* LYC4 scaffold 1459 contained both markers P8-8 and UP15 at a distance of 24 kb, whereas in *S. lycopersicum* ‘Heinz’ the distance between these two markers is 247 kb.

The three scaffolds 531, 1459 and 770 of *S. habrochaites* LYC4 were connected to form a superscaffold. To confirm the linkage between the scaffolds PCRs were performed. PCR products spanning the gaps between the scaffolds were obtained and sequenced. The gap between scaffolds 531 and 1459 proved to be small (606 bp, Fig. 1a). The gap between scaffolds 1459 and 770 was larger, approximately 4.3 kb. Thus, by closing the gaps we confirmed the orientation of the three scaffolds.

By aligning the *Ty*-*2* regions of *S. lycopersicum* ‘Heinz’ and *S. habrochaites* LYC4 we observed an inversion of ±200 kb in the central part (Fig. 1b). Within this inversion there is good co-linearity between ‘Heinz’ and LYC4, except for some gaps (unknown sequences) in the assemblies, of which the largest ones are indicated in Fig. 1a. This inversion coincides with the ‘suppression of recombination’ block in progeny of the interspecific cross between *S. lycopsersicum* and the *Ty*-*2* donor *S. habrochaites* B6013 (Yang et al. 2014).

### Recombinant screening within *S. habrochaites* species

Previously, Yang et al. (2014) reported a severe suppression of recombination in the *Ty*-*2* region in an interspecific cross between *S. lycopersicum* and a BC_4_S_2_ introgression line derived from *S. habrochaites* B6013 (donor of the *Ty*-*2* gene). Among 11,000 F_4_ plants no recombinants were observed between markers TG36 and C2_At3g52090 (Fig. 1).

**Fig. 2:**
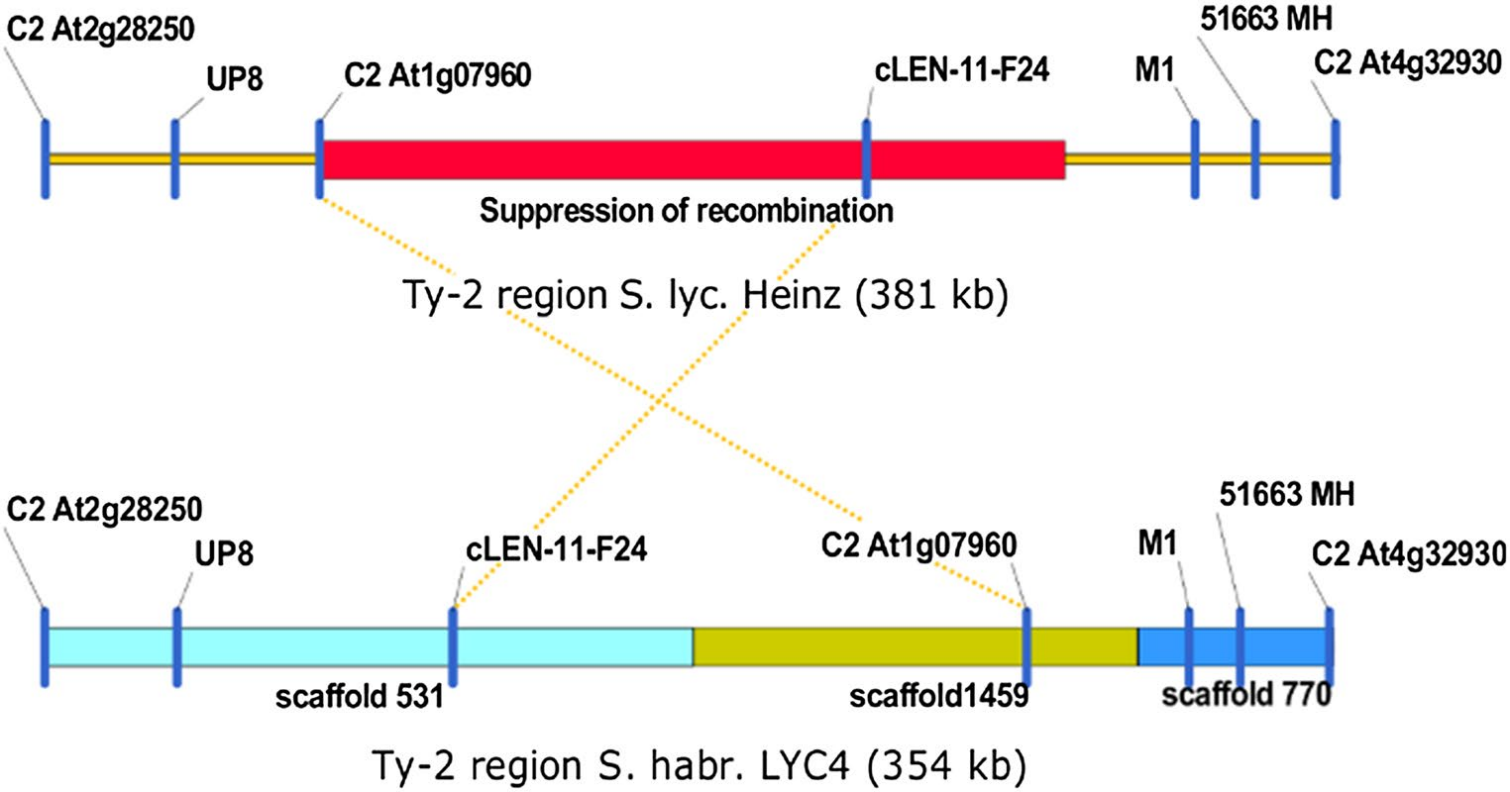
Confirmation of inversion in the *Ty*-*2* region by genetic analysis. CAPS markers used for recombinant screening of intraspecific *S. habrochaites* F_2_ populations are indicated. UP8 is included as a reference to delineate the *Ty*-*2* region, but was not used as marker

We investigated whether suppression of recombination in the *Ty*-*2* region is also occurring in intraspecific *S. habrochaites* crosses. For this, we analysed F_2_ populations of three crosses between four different *S. habrochaites* accessions. All crosses had one parent in common, which is accession G1.1560. This accession was chosen as the common parent because it shows a relatively high level of polymorphisms compared with the other three accessions that are more similar to each other. Recombinant screening was performed on 91 to 287 F_2_ progeny per cross using selected CAPS markers in the *Ty*-*2* region that had been shown to be polymorphic between G1.1560 and the other *S. habrochaites* accessions (Fig. 2). These include one marker above the ‘suppression of recombination’ block (C2_At2g28250), two markers within the block (C2_At1g07960/UF_07960 and cLEN-11-F24), and three markers below the block (M1, 51663_MH and C2_At4g329530). Markers C2_At1g07960 and UF_07960 are derived from the same gene, but amplify different fragments. Polymorphisms could be detected in one or the other marker, depending on the crossing population.

First, we analysed occurrence of recombination between markers C2_At1g07960/UF_07960 and cLEN-11-F24. The physical distance between these markers in the *S. lycopersicum* ‘Heinz’ genome is 162 kb, while the genetic distance between these markers is 4.5 cM in the Tomato-EXPEN 2000 genetic map. In total, 21 recombinants were found between these two markers (Table 1), seven in population 1 (PV960357, 91 plants), six in population 2 (PV970303, 287 plants) and eight in population 3 (PV960350, 96 plants). This indicated that there is no suppression of recombination in this region in intraspecific *S. habrochaites* crosses, although the genetic distance between these two markers varies among the crosses (2–8 cM).

**Table 1:**
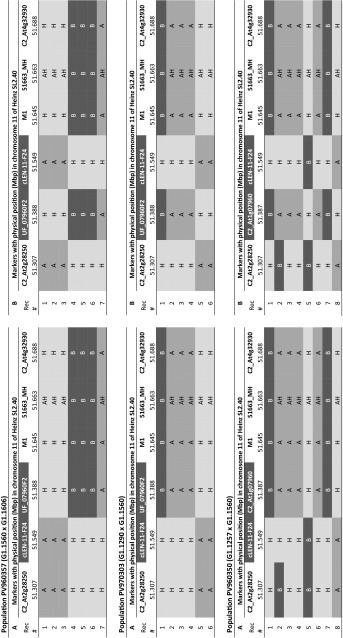
Recombinants in the ‘suppression of recombination block’ in three populations derived from crosses between different *S. habrochaites* accessions

Marker order in the A panels is based on the *S. habrochaites* LYC4 de novo sequence; markers in the B panels are ordered according to the *S. lycopersicum* ‘Heinz’ genome sequence. Markers highlighted in dark grey are located in the ‘suppression of recombination block’. Numbers under marker names correspond to their positions on chromosome 11 of tomato ‘Heinz’ genome sequence SL2.40 (in Mbp). Markers were scored in the following way: *A* homozygous for *S. habrochaites* G1.1560 allele, *B* homozygous for other parent *S. habrochaites* allele, *H* heterozygous, *AH* no distinction possible between homozygosity for G1.1560 allele or heterozygosity

To determine marker order in *S. habrochaites*, markers flanking the ‘suppression of recombination’ block (UP8, C2_At2g28250, M1, 51663_MH and C2_At4g32930) were included in the analysis (Table 1). When the markers are ordered according to the *S. lycopersicum* ‘Heinz’ genome three crossovers in a relatively small region of 338 kb are required to explain the obtained recombinant genotypes. However, a single recombination is sufficient to explain these genotypes when the order of markers C2_At1g07960 and cLEN-11-F24 is reversed. This strongly suggests that an inversion of the region containing these two markers is present in multiple *S. habrochaites* accessions compared with *S.* *lycopersicum*.

To investigate whether suppression of recombination in the *Ty*-*2* region is unique to the cross described by Yang et al. (2014) we analysed 88 F_2_ plants from a different interspecific cross, between *S. lycopersicum* ‘Moneymaker’ (MM) and TYLCV-susceptible *S. habrochaites* accession G1.1257 (parent of population 3, PV960350). No recombination events were found between markers C2_At1g07960/UF_07960 and cLEN-11-F24, suggesting a suppression of recombination in this population.

### Analysis of inversion breakpoints

**Fig. 3:**
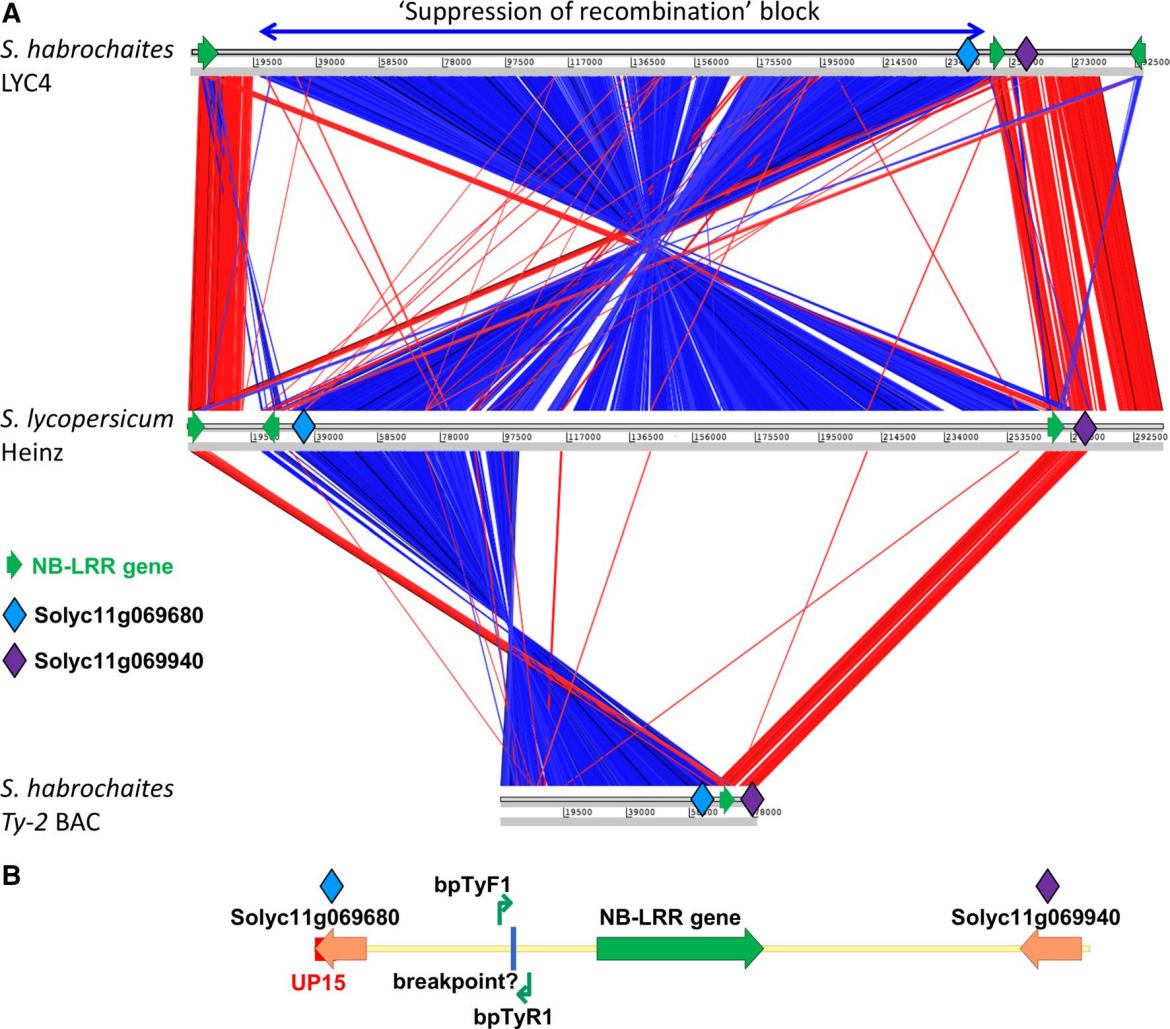
Comparative sequence analyses (**a**) Visualization of the alignment of the *S. habrochaites* LYC4 *Ty*-*2* region and the BAC sequence from the *Ty*-*2* introgression line with the *S. lycopersicum* ‘Heinz’ *Ty*-*2* region. Red lines indicate homology in the direct orientation (+ strand). *Blue lines* indicate homology in the inverse orientation (− strand). The blue lines indicate the presence of an inversion when comparing *S. habrochaites* with *S. lycopersicum*. NB-LRR gene sequences (indicated as *green arrows*) align to different positions in both direct and inverse orientation. Positions of genes homologous to Solyc11g06980 and Solyc11g069940 are indicated. **b** Part (18 kb) of the *Ty*-*2* BAC containing the lower inversion breakpoint. Primers (bptyF1 and bpTyR1) spanning the putative inversion breakpoint are indicated

So far, only a draft version of the de novo assembly of the *S. habrochaites* LYC4 genome is available. Alignment of the LYC4 superscaffold to the ‘Heinz’ genome sequence showed that the inversion was flanked by NB-LRR-like genes in inverted orientation in the ‘Heinz’ genome (Fig. 3a). One could argue that the inversion in the LYC4 superscaffold is due to misassembled sequences. To obtain evidence for the presence of an inversion in the *Ty*-*2* region in *S. habrochaites* compared with *S. lycopersicum,* a BAC library was made of a *Ty*-*2* introgression line. This line contains a small introgression of the *Ty*-*2* region from *S. habrochaites* ‘B6013′ (donor of the *Ty*-*2* gene) in an otherwise *S. lycopersicum* background. A BAC containing the UP15 marker (Fig. 1a) was obtained and sequenced. Alignment of this sequence to the *S. lycopersicum* ‘Heinz’ sequence (Fig. 3a) showed that a large part was homologous to the upper end of the inversion in *S. lycopersicum*, which is as expected based on the location of the UP15 marker which is derived from gene Solyc11g069680 (Fig. 3b). However, it additionally contained a sequence homologous to gene Solyc11g069940, which is close to M1 (Fig. 1), a marker at the other side of the inversion. Thus, the BAC contained predicted genes homologous to Solyc11g069680 and Solyc11g069940 in close proximity (18 kb) (Figure S1). In between these genes an NB-LRR type of gene is predicted that shows homology to both Solyc11g069660 and Solyc11g069930.

**Fig. 4:**
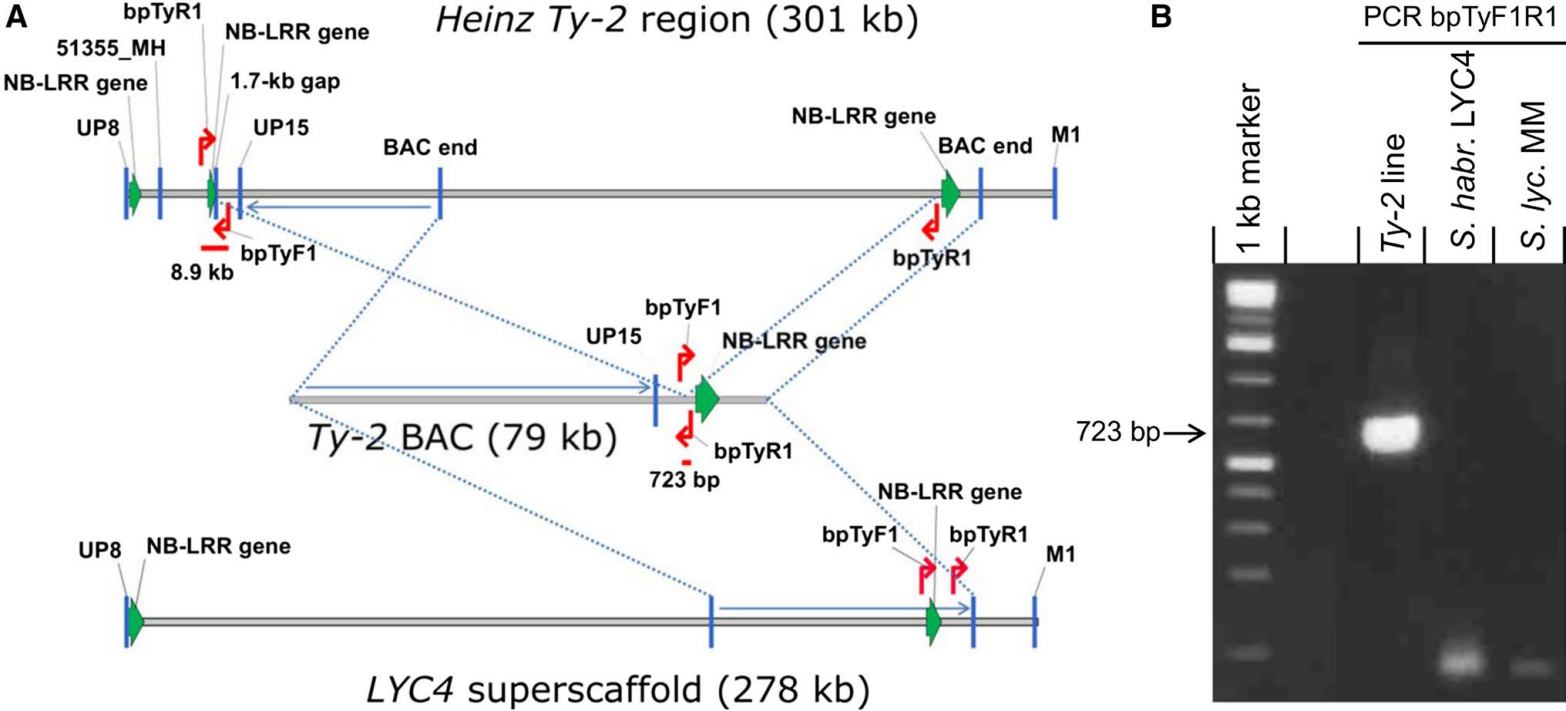
Analysis of inversion breakpoint (**a**) Location of breakpoint primers bpTyF1 and bpTyR1 (*red arrows*) flanking the inversion breakpoint in the BAC sequence obtained from the *Ty*-*2* introgression line (*middle* sequence). Location of these primers is also shown in the *S. lycopersicum* ‘Heinz’ region (*upper* sequence) and *S. habrochaites* LYC4 superscaffold (*lower* sequence). Green arrows indicate sequences homologous to NB-LRR genes. *Blue dotted lines* indicate co-linear regions. **b** PCR with primers bpTyF1 and bpTyR1 flanking the inversion breakpoint. Only in the *Ty*-*2* introgression line a PCR product is obtained

A detailed analysis of the BAC sequence was performed to determine the location of the inversion breakpoint (Fig. 4a). Primers flanking the potential breakpoint were designed on the *Ty*-*2* BAC sequence (Figs. 3b, 4a). They amplified a 732-bp fragment in the *Ty*-*2* introgression line (Fig. 4b). As expected, no PCR product was obtained with *S. lycopersicum* MM DNA. In the ‘Heinz’ sequence the reverse primer bpTyR1 is present in the same orientation as in the *Ty*-*2* BAC sequence, while the forward primer bpTyF1 is present in the inverse orientation. However, reverse primer bpTyR1 is also present on the other side of the inversion in ‘Heinz’, between markers 51355_MH and UP15. A PCR product of approximately 8.9 kb might be obtained if the adequate PCR conditions for long PCR products would be applied. Remarkably, also no PCR product was obtained for *S. habrochaites* LYC4 (Fig. 4b). When comparing the *Ty*-*2* BAC sequence with the LYC4 superscaffold sequence we found that the forward primer bpTyF1 was in the expected position above an NB-LRR gene (Fig. 4a). However, the reverse primer bpTyR1 was present below the NB-LRR gene, in the same orientation as the forward primer bpTyF1. This explains why no amplification product was obtained for LYC4.

The 732-bp sequence of the PCR product obtained from the *Ty*-*2* introgression line (Fig. 4b) was aligned with the sequence upstream of the unique primer bpTyF1 in the ‘Heinz’ genome (Figure S1A). These sequences showed a poor alignment, except for the first 154 bp starting from primer bpTyF1. In order to verify this breakpoint region in cultivated tomato, primer bpTyR2 was developed based on the ‘Heinz’ genome sequence (Figure S1B). A PCR with primers bpTyF1 and bpTyR2 resulted in the expected 687-bp product in *S. lycopersicum* MM but not in the *Ty*-*2* introgression line (Figure S1C). The sequence of the 687-bp PCR product of MM was identical to the sequence in ‘Heinz’. Although the alignment of the sequences of the two PCR products show an abrupt end of co-linearity, it is preliminary to conclude that this is the exact breakpoint of the inversion. To verify this conclusion, we need to know the sequence of the upper breakpoint region in the *Ty*-*2* introgression line. Figure S1B shows the regions harbouring the upper and lower breakpoints in *S. lycopersicum* ‘Heinz’, and the lower breakpoint in the BAC clone from the *Ty*-*2* introgression line. Gene Solyc11g069680 is present in the upper breakpoint region, while gene Solyc11g069940 is present in the lower breakpoint region in the ‘Heinz’ genome. Both genes are adjacent to NB-LRR gene fragments. The *Ty*-*2* BAC sequence contains orthologs of both Solyc11g069680 and Solyc11g069940, separated by a NB-LRR gene.

## Discussion

### Chromosomal rearrangements are frequently associated with resistance gene clusters

We report the presence of an inversion of a ±200 kb region on the long arm of chromosome 11 in *S. habrochaites* compared with *S. lycopersicum*. This inversion is different from the 294-kb inversion underlying the *fasciated* locus on the long arm of chromosome 11, which is polymorphic within the cultivated *S. lycopersicum* germplasm (Huang and Van der Knaap 2011).

There are numerous examples of chromosomal rearrangements/inversions associated with (introgression of) R-genes or R-gene clusters. Two R-gene clusters in the *Mi* locus on the short arm of chromosome 6 in *Solanum peruvianum* are separated by approximately 300 kb region, which is inverted compared to *S. lycopersicum* (Seah et al. 2007). The introgression of the *Ty*-*1* locus on the long arm of chromosome 6 from *S. chilense* in *S. lycopersicum* background shows an inversion and suppression of recombination (Verlaan et al. 2011). The *H1* locus on the distal end of chromosome 5 of potato (Finkers-Tomczak et al. 2011) shows repression of recombination in a region of at least 170 kb. The *R1* locus in the same region was shown to be present in a region that was inverted in tomato compared with potato (Achenbach et al. 2010). The donor of the *R1* gene, *S. demissum*, contained haplotypes that were highly diverged in the R-gene cluster region, while the flanking non-resistance gene regions were conserved (Kuang et al. 2005). A 70-kb inversion between the resistant R1 and the susceptible r1 haplotypes was reported by Ballvora et al. (2007). The clubroot resistance region in *Brassica rapa* has an internal inversion compared with *Arabidopsis* of about 310 kb (Suwabe et al. 2012). Suppression of recombination in these R-gene regions may be a consequence of the chromosomal rearrangement. On the other hand, suppressed recombination may also be caused by the pericentromeric position of the introgression rather than the inversion, as is the case for the *Mi*-*1* locus (Seah et al. 2007).

For other resistance gene loci suppressed recombination has been reported, but it is unknown whether this is a consequence of chromosomal rearrangements, and/or of pericentromeric locations. These include the *Tm*-*2a* gene from *S. peruvianum* introgressed in *S. lycopersicum* (Pillen et al. 1996), the *MXC3* gene in poplar, the *Lr20*-*Sr15*-*Pm1* resistance locus and *Sr22*, *Lr9*, *Lr24* and *Lr35* resistance genes in wheat, the *Mla* and *Mlg* powdery mildew resistance gene clusters and the *Rrs2* resistance gene in barley (reviewed in Hanemann et al. 2009), and the *Rhg1*/*Rfs2* locus in soybean (Afzal et al. 2012).

Chromosome rearrangements complicate the fine-mapping and cloning of resistance genes, especially when they involve large regions containing many genes. In the case of the *Ty*-*2* resistance gene it was shown previously that it is unlikely to be a typical NB-LRR gene, because silencing of the NB-LRR candidates in the *Ty*-*2* region did not result in compromised TYLCV resistance (Yang et al. 2014).

### Advantage of de novo genome assemblies of wild relatives of crop species

After the assembly of the genome of cultivated crop species the focus has shifted to sequencing related wild species at low read depth to obtain information on sequence variation by mapping reads to the reference genome. The assumption is that there is a high degree of co-linearity within a species and between closely related species, and that a large set of SNP markers developed after re-sequencing can be used to fine map traits of interest. However, as shown by Huang and van der Knaap (2011) chromosomal rearrangements may occur even within a cultivated species. Re-sequencing data consisting of small reads do not provide positional information of SNP markers, or SNP marker order. Therefore, such data do not uncover the presence of chromosomal rearrangements in wild species, especially those that are not closely related to the cultivated species as shown in tomato (Szinay et al. 2012). FISH using BAC clones has been demonstrated to be a powerful tool in the study of chromosomal rearrangements (Lou et al. 2010; Verlaan et al. 2011; Peters et al. 2012; Szinay et al. 2012; Shearer et al. 2014). However, in the *Ty*-*2* region, FISH was not successful due to the small size of the inversion (Yang et al. 2014). In this study, we show that a de novo genome assembly has been very helpful to analyse the chromosomal structure of a wild species, which can be exploited to explain unexpected recombination phenomena in crosses with the cultivated species.

Also within a wild species there may be accessions that show small-scale rearrangements, as we observed when comparing the inversion breakpoint between *S. habrochaites* LYC4 and the *Ty*-*2* BAC sequence derived from *S. habrochaites* B6013. Therefore, BAC libraries may still be required to zoom in on the gene of interest in specific accessions.

### Perspectives for resistance gene cloning

Introgression of the smallest possible DNA fragment containing the gene of interest from a donor species into the crop species is often a time-consuming process, and the success can be limited when chromosomal rearrangements exist in related species used for interspecific crosses. Since genome structure and genomic co-linearity of the introgressed region between donor species and recipient crops are often unknown, breeders are ‘blind’ and cannot foresee complications in their introgression breeding programs. With the example of the *Ty*-*1* gene (Verlaan et al. 2011) and the *Ty*-*2* gene in this study, we demonstrated that FISH and genomic approaches can be applied to investigate chromosomal rearrangements in genetic mapping and introgression breeding. Furthermore, the occurrence of chromosomal rearrangements stresses the importance of a de novo genome assembly when wild *Solanum* species are sequenced.

The fact that *Ty*-*2* is located in a chromosomal region which is inverted in *S.* *habrochaites* compared with *S. lycopersicum* has consequences for the strategy of cloning this gene. For marker-assisted breeding it is not necessary to clone the gene conferring resistance to TYLCV, because linked markers in the ‘suppression of recombination’ block do not show segregation in the progeny. However, this large block introgressed from *S. habrochaites* contains at least 35 genes (Yang et al. 2014), of which it is unknown whether they have an adverse effect on plant growth, performance and yield in diverse growing conditions. Negative effects on agronomic and quality traits have been observed to be associated with introgression from the *Tm*-*2a*, *Sw*-*5* and *Ty*-*1* virus resistance genes (Rubio et al. 2012), probably due to linkage drag.

Here, we show that recombination in the *Ty*-*2* region is occurring in intraspecific crosses between different *S. habrochaites* accessions. Therefore, in order to further fine-map the TYLCV resistance gene we are generating F_2_ progenies from a cross between resistant *S.* *habrochaites* accession B6013 and susceptible *S. habrochaites* accessions that show enough polymorphisms for efficient and detailed recombinant screening. In the near future, the fine-mapped position of the *Ty*-*2* gene will show whether it is located in the inversion. If the gene is outside the inversion, it should be possible to eliminate the inversion in an introgression line carrying the *Ty*-*2* gene.

#### Author contribution statement

AMAW, YD and YB designed the experiments; AMAW, MC, SD, RF, JG and XW performed the experiments; AMAW, MC and YB analysed the data. AMAW, SD and YB wrote the paper; RGFV critically read and improved the paper.

## Electronic supplementary material

Supplementary material 1 (DOCX 123 kb)

Supplementary material 2 (PDF 34 kb)
