# Supplementary material for: Detection of an inversion in the Ty-2 region between S. lycopersicum and S. habrochaites by a combination of de novo genome assembly and BAC cloning
Source: Theor Appl Genet. 2015 Jul 8;128(10):1987–97. doi: 10.1007/s00122-015-2561-6 (PMC4572051; doi:10.1007/s00122-015-2561-6)

**Fig S1** Comparison of sequences in the breakpoint regions of the *Ty-2* inversion (**a**) Sequence alignment of the PCR products obtained with primers bpTyF1 and bpTyR1 in the *Ty-2* line, and with primers bpTyF1 and bpTyR2 in *S. lycopersicum* ‘Heinz’. Identical nucleotides are highlighted in yellow. The sequence in cultivar Moneymaker (MM) is identical to the ‘Heinz’ sequence. (**b**) Graphical representations of the upper and lower breakpoint regions in the *S. lycopersicum* ‘Heinz’ genome, and of the lower breakpoint region in the BAC sequence from the *Ty-2* line. (**c**) PCR products obtained with primers bpTyF1 and bpTyR1 in the *Ty-2* line and with primers bpTyF1 and bpTyR2 in *S. lycopersicum* Moneymaker (MM).

**A**

**bpTyF1 🡪**

bpTyF1+R1_Ty2line aaactcacaccgctccgttgtcattcctatcttccattgatttttattagatttggttgt

bpTyF1+R2_Heinz aaactcacaccgctccgttgtcattcctatcttccattgatttttattagatttggttgt

bpTyF1+R1_Ty2line ataattaagaggagcaagtttgtcaggatgtaggttggattgataacaattgaacttaat

bpTyF1+R2_Heinz ataattaagaggagcaagtttgtcatgaggtaggttggattgataacaattgaacttaat

bpTyF1+R1_Ty2line caatttcttttggcacaaacttgttatgtcttggggcc--cgtttggatgggcttaataa

bpTyF1+R2_Heinz caatttcttttggcacaaacttgttatgtcttggtcccatgctttagtccttttccatta

bpTyF1+R1_Ty2line aagcagctttaaaaaagtacttttgaaagtgctgaaatttatcatttaaagtgtatgaca

bpTyF1+R2_Heinz cattaataacccaacaacaaatatagaa----tagagcacatggttgcaaaacttatata

bpTyF1+R1_Ty2line atgtggctcggcaggattcgtttgacatccaagacagtgaagagcttaatagaatgaagg

bpTyF1+R2_Heinz ctaactctgttcaatttaagttatcggtttatttgagcacaaagtttaaaaaaaaagaag

bpTyF1+R1_Ty2line aagaaaatgttaatctacaagaaaagctgaacgatgcaggtatacattcttaattgatat

bpTyF1+R2_Heinz agatttatg-aaatttatgattaatttaaaattgcgtggtttttcaaattttgtagtgac

bpTyF1+R1_Ty2line t----acaaacactacaaatttgtaaaatatgaagaaaaaaaaaaaacggtgtgagccat

bpTyF1+R2_Heinz tcacgaggtagaatataggaattgaaaaacttacaatgaaaagtaagttttaagtgtctt

bpTyF1+R1_Ty2line atatgata------tattgaattttaaacttcttcacttgcagttatcactttcttagtc

bpTyF1+R2_Heinz acttaataatattctagtgagttaaaatattaaacgtttgtagct-----tttgctaaca

bpTyF1+R1_Ty2line aaataaaaccatagtatttcattacttgtccacaggaatcattcatgtacaactatatag

bpTyF1+R2_Heinz cattattactatagcaatt--atatgcacctattattatctcccaaaacaagatctgcca

bpTyF1+R1_Ty2line agagatgtgtgtgctttggtttgtaattgcaatatgtggattgaccaagagtttcaaaaa

bpTyF1+R2_Heinz ctaaacat-tgagcttcgttacacgataaagacatcttagtacacaaaatat--------

bpTyF1+R1_Ty2line ttcattttaattgcattcagaaaggaggttgtgatttccaagtcgtacaactgtccactg

bpTyF1+R2_Heinz tccacgttagtatggtttgagaaaaggtgcacctcaagtacttaataaaaatattagtat

bpTyF1+R1_Ty2line tgccttccttgaggtgaagcatgacttgctgcaaatgggacatctagctctagtgtaccc

bpTyF1+R2_Heinz atgctttttcacgactcgaatatattacctataagtcacatgccaacatattcatgatat

**🡨 bpTyR2**

**🡨 bpTyR1**

bpTyF1+R1_Ty2line aaagatcggaagagg

bpTyF1+R2_Heinz gatagtcccttttaa


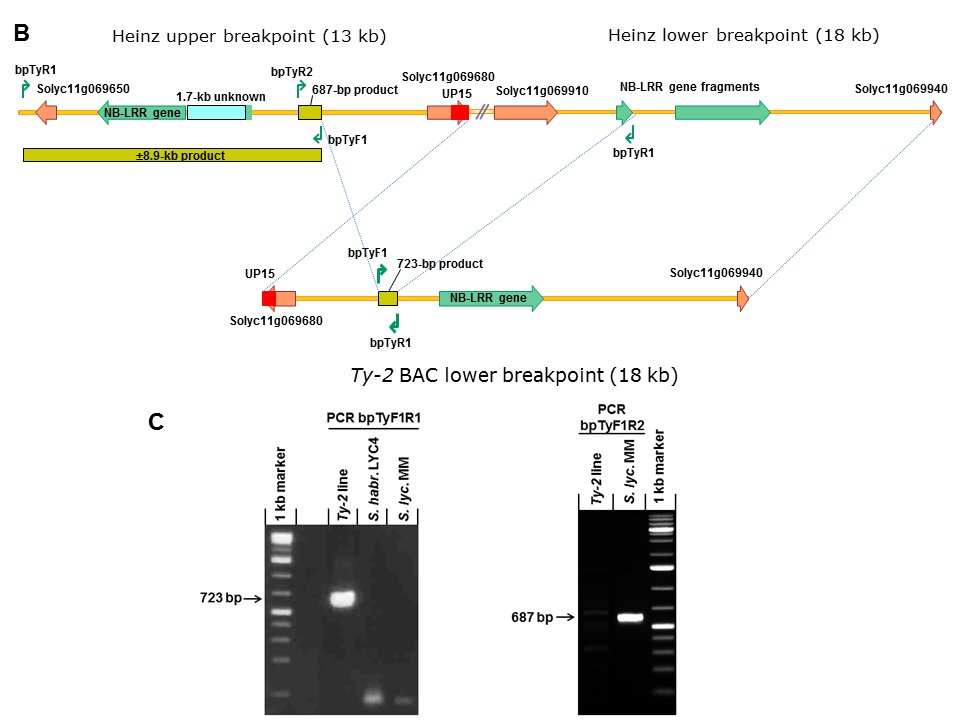

Supplement: Supplementary file 1 — Supplementary material 1 (DOCX 123 kb) [file 122_2015_2561_MOESM1_ESM.docx]
