# Supplementary material for: Detection of an inversion in the Ty-2 region between S. lycopersicum and S. habrochaites by a combination of de novo genome assembly and BAC cloning
Source: Theor Appl Genet. 2015 Jul 8;128(10):1987–97. doi: 10.1007/s00122-015-2561-6 (PMC4572051; doi:10.1007/s00122-015-2561-6)
Supplement: Supplementary file 2 — Supplementary material 2 (PDF 34 kb) [file 122_2015_2561_MOESM2_ESM.pdf]

Detection of an inversion in the *Ty-2* region between *S. lycopersicum* and *S. habrochaites* by a combination of *de novo* genome assembly and BAC cloning

Theoretical and Applied Genetics

Anne-Marie A. Wolters, Myluska Caro, Shufang Dong, Richard Finkers, Jianchang Gao, Richard G.F. Visser, Xiaoxuan Wang, Yongchen Du, Yuling Bai

Corresponding author:
Yuling Bai, Wageningen UR-Plant Breeding, Wageningen University & Research Centre, P.O. Box 386, 6700AJ Wageningen, The Netherlands
bai.yuling@wur.nl

Table S1 Primers used for recombinant screening

**Marker**	**Physical position on SL2.40ch11 (Mbp)**	**Restriction enzyme**	**Primers**
C2_At2g28250[a]	51.307	—	F-AGACTTCATCATCGTCATGTGGTTCCG<br>R-TTTGGAGGTGCTTTGCCATACCAAG
C2_At1g07960	51.388	*RsaI*	F-AAAGCCATTGTTACCGTCTCCGTG<br>R-AGCCATAAGTGGTGTGGAGGACTT
UF_07960F2	51.388	*BanI*	F-CGTGCCACCCCTTCATAATA<br>R-CCCTTGCGAGGAAAATACAG
cLEN-11-F24	51.549	*RsaI*	F-TTATGGACAGCATGGTCCTCGGAA<br>R-GAAGTCTGGGAGCGATAGTAGTCT
M1	51.645	*SsiI*	F-CGCTCGGGCAAATAGTTCGTAATGG<br>R-TTCATGGTCTAGAAATGTCCCCTGT
51663_MH	51.663	*Hin6I*	F-CCCTCTTGCTTAGTGGGTGA<br>R-ACGCTCCAAATCAGAGGTTG
C2_At4g32930	51.688	*SspI*	F-TCCTCTTCCTATTGGCAAGGGC<br>R-TGGACACTCCCCCTTTTCATCATAC

[a] SNP marker; PCR product sequenced

Table S2 Primers used for BAC clone screening

Marker	Physical position on SL2.40ch11 (Mbp)	Primers
UP8	51.345	F-GCGCTGCTAGACATTTCGAT<br>R-CTGAAGTTGCTTGAATGCTCA
UP15	51.382	F-TCTCAAAGCGTTGATCGTTG<br>R-GCTTGCTCTTGTTGGTCTCC
C2_At1g07960	51.388	F-AAAGCCATTGTTACCGTCTCCGTG<br>R-AGCCATAAGTGGTGTGGAGGACTT
D1-3	51.399	F-GGGGTGTGGTTCTCTTTGCGTT<br>R-GCCGGTACTTGCGAGCTTCTTC
D1-2	51.414	F-TGTCTGTTGTCTCTGACCCGTA<br>R-ATTCCACACTTCTCATCCCTCC
P1-16	51.427	F-CACACATATCCTCTATCCTATTAGCTG<br>R-CGGAGCTGAATTGTATAAACACG
D8-g40145	51.441	F-CCCCTATTGTTTTCTCTGTT<br>R-CCCATGTCCTATAATTTGTC
D7-g60068	51.461	F-TTGGTGGTGTGTTCTGTTTA<br>R-CGTTAGGTGGAGTAGGTGCT
D2-3	51.490	F-ATAACTGCATGGGAAGACCG<br>R-CTCCGTAAGCAACCGAAGAC
D3-2	51.503	F-TCAAAGGACGAGATACAATC<br>R-AATCAACAAAGGCTTAACAG
D4-3	51.549	F-TACTTGACCCTGCTGTTATT<br>R-GTCTGGGAGCGATAGTAGTC
D5-2	51.586	F-ATCACTTCCTTCACCCGTAA<br>R-CTCCGATTTCAACTCCATTT
C2_At3g52090	51.606	F-AGGGATACGAAGATCATGAATGCAGC<br>R-ACTCTTCAGATGATCAAGTTCCTTGTC
S1-2	51.615	F-AAATGTAGTTGATAGAAGGG<br>R-ATGCTAAAAGGTAAGGAGGT
S1-3	51.633	F-AAGGAGAAAAACGGAAGAGC<br>R-CTACAATAGCCACAGGGTCA
S1-4	51.645	F-GAAAAGGAAGTGTTGGAACA<br>R-CAATCTCATATAAATGGGGA
M1	51.645	F-CGCTCGGGCAAATAGTTCGTAATGG<br>R-TTCATGGTCTAGAAATGTCCCCTGT
